# What is the role of cardiac MRI in the diagnosis of left ventricular non-compaction?

**DOI:** 10.1186/1532-429X-15-S1-P157

**Published:** 2013-01-30

**Authors:** J Ronald Mikolich, John Lisko, Nicholas C Boniface, Brandon M Mikolich

**Affiliations:** 1 Northeast Ohio Medical University, Rootstown, OH, USA; 2 Sharon Regional Health System, Hermitage, PA, USA

## Background

The prevalence of left ventricular non-compaction is not well established. Using 2-D echocardiography, less than 1% of patients are diagnosed with this entity. Using cardiac MRI, approximately 3% of patients have evidence of LV non-compaction. This variance may be related to the difference in spatial resolution between these 2 imaging techniques. Using a large institutional cardiac imaging database, 2-D echo and cardiac MRI results were compared in patients with LV non-compaction.

## Methods

Of 1,255 patients in our cardiac imaging database, 38 patients (3%) with a diagnosis of LV non-compaction were identified. Twenty-two of these 38 patients had undergone both 2-D echo and cardiac MRI exams. The reported diagnoses with each imaging modality were tabulated, along with measurements of left ventricular antero-septal and infero-posterior wall thickness for both modalities. The 2-D echo and cardiac MRI wall thickness dimensions were statistically compared using a paired sample t-test.

## Results

All 38 patients had a diagnosis of LV non-compaction on cardiac MRI, using criteria of non-compacted/compacted myocardium > 2.5 to 1.0 and deep LV trabeculations. None of the 22 patients with both 2-D echo and cardiac MRI exams had a diagnosis of LV non-compaction on their 2-D echo study. However, 15 of 22 patients (68%) had a diagnosis of LVH on their 2-D echo study, while only 3 had LVH on their cardiac MRI study. The mean ASWT on 2-D echo was 1.38 cm, while the mean ASWT on cMRI was 1.16 cm (p<0.005). The mean IPWT on 2-D echo was 1.31 cm, while the IPWT on cMRI was only 0.89 cm (p<0.001).

## Conclusions

These data suggest that LV non-compaction, identifiable on cardiac MRI, is frequently diagnosed as LVH on 2-D echo. This discrepancy appears to be explained by the statistically significant difference in both antero-septal and infero-posterior LV wall thickness measurements between the 2 imaging modalities. It is suspected that this difference is related to the inferior spatial resolution of 2-D echo relative to cMRI. On the basis of these data, patients with LVH on 2-D echo should be considered for a cardiac MRI exam to determine if the increased LV wall thickness is truly LVH or an undiagnosed LV non-compaction cardiomyopathy.

## Funding

Not Applicable. None.

